# On the Role of Processing Parameters in Producing Recycled Aluminum AA6061 Based Metal Matrix Composite (MMC-Al_R_) Prepared Using Hot Press Forging (HPF) Process

**DOI:** 10.3390/ma10091098

**Published:** 2017-09-19

**Authors:** Azlan Ahmad, Mohd Amri Lajis, Nur Kamilah Yusuf

**Affiliations:** Sustainable Manufacturing and Recycling Technology, Advanced Manufacturing, and Materials Center (SMART-AMMC), Universiti Tun Hussein Onn Malaysia, 86400 Parit Raja, Batu Pahat, Johor, Malaysia; amri@uthm.edu.my (M.A.L.); nurkamilahyusuf@gmail.com (N.K.Y.)

**Keywords:** sustainable manufacturing, direct metal recycling, hot press forging, aluminum recycling, aluminum AA6061, reinforcement particles

## Abstract

Solid-state recycling, which involves the direct recycling of scrap metal into bulk material using severe plastic deformation, has emerged as a potential alternative to the conventional remelting and recycling techniques. Hot press forging has been identified as a sustainable direct recycling technique that has fewer steps and maintains excellent material performance. An experimental investigation was conducted to explore the hardness and density of a recycled aluminum-based metal matrix composite by varying operating temperature and holding time. A mixture of recycled aluminum, AA6061, and aluminum oxide were simultaneously heated to 430, 480, and 530 °C and forged for 60, 90, and 120 min. We found a positive increase in microhardness and density for all composites. The hardness increased approximately 33.85%, while density improved by about 15.25% whenever the temperature or the holding time were increased. Based on qualitative analysis, the composite endures substantial plastic deformation due to the presence of hardness properties due to the aluminum oxide embedded in the aluminum matrix. These increases were significantly affected by the operating temperature; the holding time also had a subordinate role in enhancing the metal matrix composite properties. Furthermore, in an effort to curb the shortage of primary resources, this study reviewed the promising performance of secondary resources produced by using recycled aluminum and aluminum oxide as the base matrix and reinforcement constituent, respectively. This study is an outline for machining practitioners and the manufacturing industry to help increase industry sustainability with the aim of preserving the Earth for our community in the future.

## 1. Introduction

Conventional methods of aluminum recycling usually use the melting of aluminum scrap and then manipulate the molten aluminum through a manufacturing process so that it can be used again. Accordingly, managing the energy consumption within the industrial process has become eminent in the industrial sector. Despite the high energy consumption in the recycling process, there is no assurance that the aluminum will be completely recycled. Metal is lost in every stage of the recycling process. The losses have been attributed to metal oxidation during melting and burning, jumbled metal and slag from the surface of the melt, and scrap creation due to casting and further processing of aluminum ingots [1]. Many studies have discussed and concluded that, when compared to alternative recycling techniques such as powder metallurgy, extrusion, or forging, conventional recycling leads to higher costs and energy consumption, and has a negative environmental impact [2,3].

Although metal recycling has many advantages, it has not been spared from environmental dispute. In recent years, many attempts have been made to create environmentally-friendly recycling technologies that are adaptable, reliable, energy saving, free from harmful substances, and reduce production waste and harmful emissions [4,5]. Direct conversion methods of recycling aluminum have many benefits, including cost and energy savings, and environmental protection by reducing the hazardous gas emissions compared to traditional recycling methods [1]. Hot press forging (HPF) is a new alternative direct recycling approach which has fewer steps, lower energy consumption, and is cost-effective due to the process operating above the recrystallization temperature [6]. The HPF process eliminates the two traditional intermediate processes of cold-compacting and pre-heating, which leads to lower energy consumption without interfering in the metallurgical processes.

While secondary aluminum exhibits superior mechanical properties, it tends to fail more often than primary aluminum. Consequently, ceramic materials are used to enhance the alloys’ mechanical properties, and the resultant combination is called a metal matrix composite (MMC). Like other composites, the creation of MMCs include at least two physically and chemically distinct phases. For matrix alloys, the combination of a ductile material with a high strength material, to reinforce the constituent, leads to notable shear and compression strength, and superior service–temperature abilities [7,8,9,10]. Compared to unreinforced alloys, all reinforced forms have significantly better retention of anisotropic properties, and specific properties at room and high temperatures [11,12,13,14]. Despite encouraging results with the reinforcements, there were still defects in the composites, mostly during the fabrication process (i.e., particles congregated severely, irregular voids from rapid coagulation, and cylindrical voids precipitated by dissolved gases). On the other hand, for particulate reinforced MMCs, reinforcements have been shown to be beneficial. The particle reinforced constituents should be further studied because they are inexpensive, they have promise for manufacturing processes for MMC development, and metal working methods to form particulate MMCs are simple [10,15].

However, there are also studies that have been done to investigate the machining process for hard-to-machine material like MMCs. Conventional machining processes of MMCs are often related with heavy tool wear and poor surface finish, due to the hard ceramic reinforcement of the material [16,17]. Previously, laser assisted machining process was found to show considerable improvement in machinability of MMCs [18]. Thus, heating the material during cutting was suggested in order to improve the machining properties of such material. Through proper manufacturing processes, MMCs were acknowledged for many uses. The transportation sector most commonly uses MMCs due to their high strength-to-weight ratio and mechanical and thermal properties. Aerospace structures could also benefit from the high stiffness and strength features of the material as it reduces the probability of buckling. Additionally, MMCs are also used to manufacture connecting rods through hot pressing. The technique is practical and the corresponding waste is minimized as a result of using the near-net shape process [7].

Despite various recent advances in the fabrication of MMCs, the use of such advanced processes has been limited to date. Producing a satisfactory end product is difficult, especially when the machining process can cause surface damage which can lead to change in the product properties, such as fatigue and corrosion resistance [19]. Hence, the HPF process is better for near-net-shape components to avoid damage to the composite. HPF can also improve the anisotropic properties through work-hardening the matrix and enhancing the microstructural features, such as reducing porosity [7,19]. The microstructure becomes finer by increasing the operating temperature, holding time, and appropriate flow stress. The properties of the material can be altered by varying the process parameters. HPF exerts sufficient pressure to the composite for better bonding, and thus improves the material’s performance.

This study intended to investigate the influence of HPF processing parameters on the microhardness and density of recycled aluminum-based metal matrix composite (MMC-Al_R_). This study used reinforced recycled aluminum waste and introduces an innovative approach for recycling that uses HPF with fewer processing steps. In previous studies, the effects of aluminum oxide (Al_2_O_3_), as a reinforced particle, on the integrity of the recycled specimen of AA6061 aluminum alloy—particularly in HPF operation—have not been evaluated [20,21]. Thus, an investigation into the potential uses of aluminum waste material was required.

## 2. Materials and Methods

Aluminum alloy 6xxx series was obtained, and the certificate verified the bulk to be 6061 as-received (AR). Several tests and analyses were completed to obtain rudimentary data on the alloy behavior. The bulk was cut, ground, and prepared for the microhardness test, density test, structural evolution analysis, and element analysis. The material properties are presented in Table 1.

**Figure 1:**
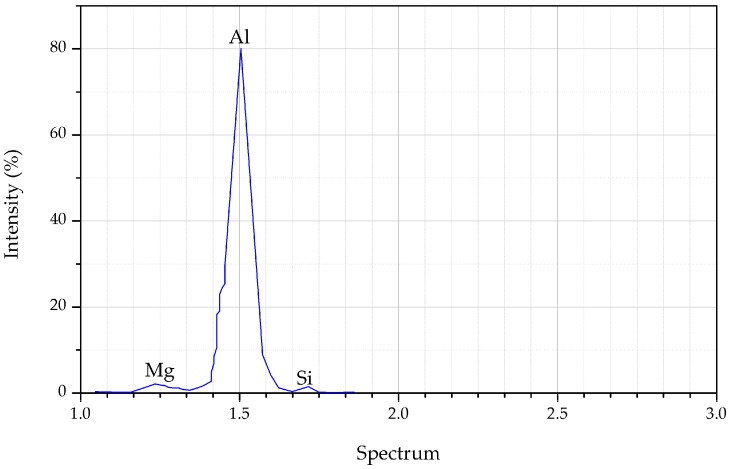
EDS spectra for AA6061.

A uniform cubical sample was cut from the bulk aluminum. The sample was ground and polished until the surface was flat and smooth, which is necessary for X-ray analysis. Localized chemical analysis was attained from the X-ray spectrum emitted by the solid sample. The energy-dispersive X-ray spectroscopy (EDS) was obtained from a Hitachi SU1510 (Tokyo, Japan) and the amounts of each element were obtained from the surface of the cubical sample. We determined the 6xxx series aluminum consisted of Al-Mg-Si in a balanced composition. As shown in Figure 1, aluminum was the most common element, followed by Mg (0.92 wt %), Si (0.58 wt %), and other elements (<0.01 wt %).

### 2.1. Preparation

The AA6061 bulk was milled using Sodick-MC430L high speed machining (Baginton, Coventry, UK). The chips were produced by manipulating the machining parameters. Medium-sized chips yielded better specimen performance and the machining parameters are shown in Table 2 [21,22].

The chips were cleaned as soon as they left the milling machine. A 99.5% pure acetone (C_3_H_6_O) (Sigma-Aldrich, Saint Louis, MO, USA) was used to clean the chips, and then they were dried for about 30 min in a thermal oven at a temperature of 60 °C [23]. Next, the aluminum AA6061 was mixed with Al_2_O_3_ powder. The process of mixing both materials was performed in a three-dimensional (3D) mixing machine (Xiongshun, Shanghai, China). The AA6061 aluminum chips were mixed with 2.0 wt % Al_2_O_3_ powder.

### 2.2. Hot Press Forging Process

The next step used the hot press to forge the chips into shape. This process was conducted for 60, 90, and 120 min, while the temperatures were set to 430, 480, and 530 °C. The experimental parameters and specimen designation are shown in Table 3.

Twelve grams of cleaned, dried, and mixed aluminum chips were poured into the mold and the plunge was fixed. The mold was then placed inside the HPF machine to complete the forming operation.

**Figure 2:**
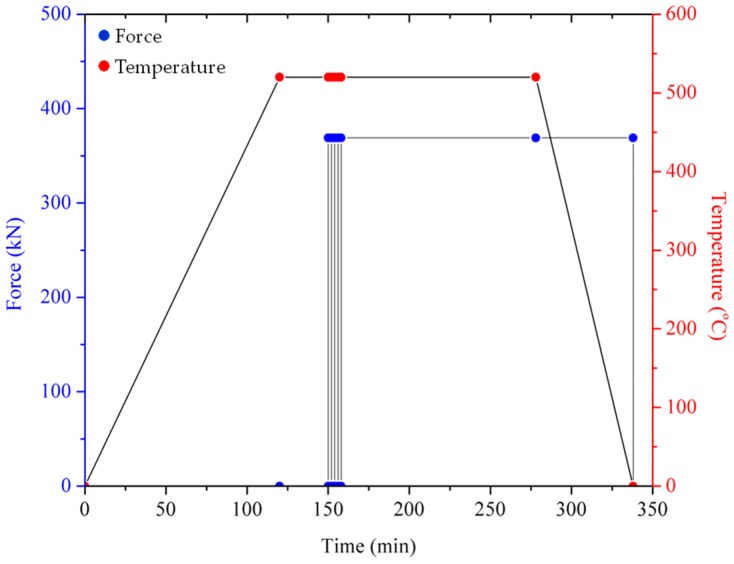
Force and operating temperature over time.

A graph of the HPF operating cycle is shown in Figure 2. For the first 120 min, the mold is heated and when it reaches the desired temperature, the holding stage occurs for 30 min to stabilize and uniformly distribute the heat into the entire working area. The temperature is maintained during the pre-compacting cycle (PCC) and the plunge is repeatedly pressed with 350 KN force for several cycles. The temperature and force are constant until the end of the soaking time. Finally, the heat is turned off, but the plunge remains steady at maximum force to begin the cooling stage.

### 2.3. Hardness Test

Microhardness (MH) is the testing of a material’s hardness using small applied loads. Vickers hardness test was used by applying force into the surface of the composite using a square-based pyramidal diamond indenter with specified face angles, under a predetermined force of 2.942 N (HV_0.3_) for 10 s [24]. Ten indentations were applied to the specimen, and each indentation distance was constant at 2.0 mm. The diagonals of the resulting impression were measured after removal of the force.

### 2.4. Density Measurement

The experimental density of the composites was obtained by the Archimedian method by weighing small pieces cut from the composite, first in air and then in water. The amount of porosity was then determined by comparing the theoretical density with the measured density determined with the Archimedes method. The theoretical densities were calculated using the mixture rule according to the weight fraction of the aluminum oxide particles [25,26].

(1) $$\;\rho_{c}=\left(\rho_{m}\times f_{m}\right)+\rho_{r}\left(1-f_{m}\right)$$

where *ρ_c_*, *ρ_m_*, and *ρ_r_* are the theoretical densities for the composite, matrix, and reinforced constituent, respectively, and *f_m_* is the volume fraction of the matrix.

### 2.5. Crystal Structure Analysis

X-ray diffraction (XRD) patterns were obtained and analyzed for AR and the reinforced milled chips at both the minimum and maximum parameter settings. The patterns were recorded using Bruker D8 Advance (Billerica, MA, USA) with Cu Kα radiation (*λ* = 1.54060 nm) at 40 kV and 40 mA settings. The 2-theta (2*θ*) ranges logged for all samples were 30–90°. The diffraction peaks were analyzed using standard XRD procedure to determine the crystal structures and lattice parameters of the phase. The instrumental broadening ($\beta_{i}$) was removed by using a diffraction pattern from the line broadening of a standard material, such as annealed Si powder [27,28]. The sizes were obtained through EVA software which helps to solve diffraction problems. By comparing the pattern of all the samples, we were able to identify the phases present in each sample.

## 3. Results and Discussion

Table 4 shows the responses obtained with Vickers hardness and density with respect to different temperature and holding times for each MMC-Al_R_ as designated in Table 3. Each of the Vickers hardness values is an average value from 10 indentation readings obtained from each sample. The value shown for the MMC-Al_R_ density is an average from five repetitions of the density measurement for each specimen. Both results show an enhancement of MMC-Al_R_ performance for each temperature and holding time increase.

### 3.1. Vickers Microhardness

**Figure 3:**
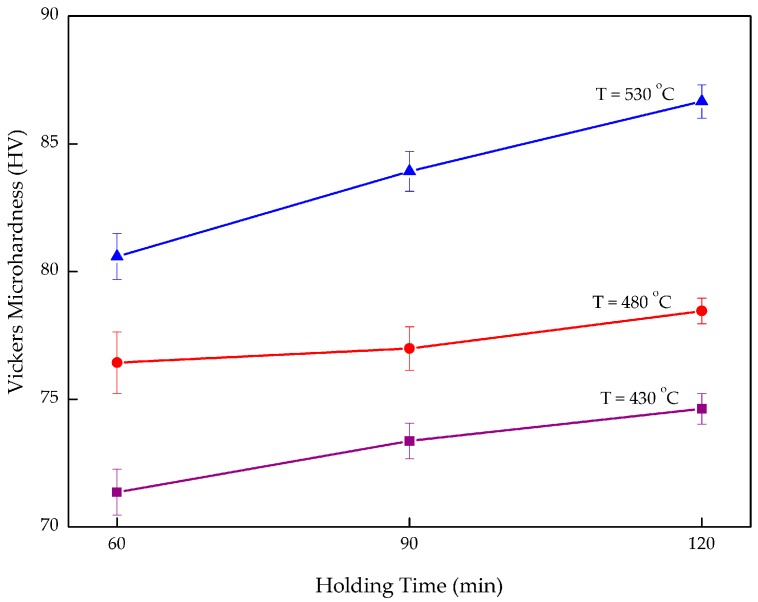
Microhardness responses in relation to different temperature and holding times.

The average measurements of the indentations are depicted in Figure 3. The microhardness value grew as the operating temperature and the holding time were increased. By increasing the operating temperature from 430 to 530 °C at a constant holding time of 90 min, the microhardness increased by 14.39% from 73.367 to 83.921 HV_0.3_. Subsequently, a similar trend was identified when studying the effect of holding time at the same temperature (530 °C). The microhardness increased 7.52% from 80.592 HV_0.3_ after 60 min, to 86.656 HV_0.3_ after 120 min. This positive trend also applies to all the specimens whenever the temperature or the holding time is increased. In short, the operating temperature is the most influential factor for increasing microhardness.

The analysis of grain structure determined that the grain average diameter reduces as the temperature and holding time increase, as shown in Table 5. Size reduction is one of the factors influencing the increase in hardness. The relationship between grain size and composite performance has been previously discussed [29].

The grain growth of the composite is suppressed by the ceramic additive which improves the hardness. Several studies have revealed that, according to the Hall–Petch rules, a fine grain size is beneficial for obtaining high microhardness [29,30,31,32,33,34]. In addition, the hardness of Al_2_O_3_ embedded in soft aluminum can be attributed to the increase in the composite hardness. According to Al-Mosawi et al. [35], the presence of sub-micrometric Al_2_O_3_ particles has caused a dispersion strengthening mechanism that may enhance the material toughness. The presence of a reinforcement constituent limited the localized matrix deformation due to increasing the intermetallic phase during the forming process [36,37]. Subsequently, the hard ceramic particles of Al_2_O_3_, which have a higher hardness value compared to the ductile matrix, allowed the matrix to endure large plastic deformation [38]. Likewise, Rahimian et al. [39] stated that the greater interfacial area between the hard (Al_2_O_3_) and soft phase (AR) is the reason for the increase in the composite’s hardness. This was proven through the increase in the hardness of the reinforced aluminum. Moreover, as the temperature and holding time increased, the matrix had high kinetic energy which leads to higher solubility limits and the formation of intermetallic bonding. The matrix itself assisted in retaining the composite hardness.

**Figure 4:**
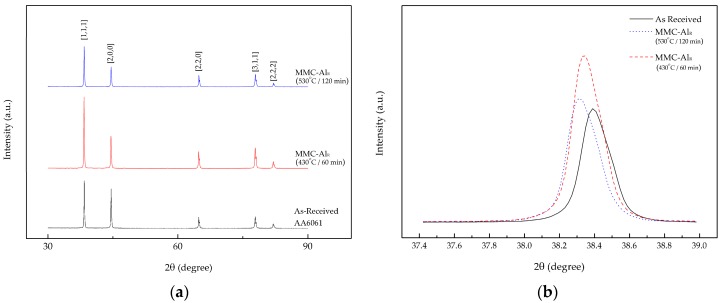
X-ray diffraction (XRD) pattern for as-received (AR) and metal matrix composite (MMC-Al_R_) at minimum and maximum parameter settings for the (**a**) whole spectra and (**b**) peak (1,1,1).

In addition, the XRD analysis showed agreement with the composite behavior. Figure 4a shows the XRD pattern for the three different materials. All the major peaks in the pattern are identified as belonging to aluminum, with a face-centered cubic crystal structure and a lattice parameter of a = 0.4049 nm. XRD spectra were validated with the standard Aluminum (JCPDS file No. 00-045-1204). The alloy peaks were observed to match along 2-theta of 30 to 90 degrees with the standard, suggesting the alloy is an aluminum series. The peak height for the first peak was reduced when the temperature and holding time increased from 430 to 530 °C and 60 to 120 min, respectively (Figure 4b, Table 6). The d-spacing also decreased, which in turn resulted in a tapering line of the peak for the maximum parameter setting. This line tapering of the composite at 530 °C and 120 min resulted in an increase in the crystallite size from 739.9 to 770.6 Å. When compared to the XRD pattern of the maximum parameter setting composite with AR AA6061, the crystallite size is much smaller. This is due to the AR sample is in isotropic condition and the structure does not react to temperature or pressure in comparison to the recycled composite. We also noticed that the position of the peaks shifted slightly (Figure 4b). The shifted peaks could be related to the dissolution of impurities, particularly iron, in the lattice of aluminum [27]. The shifted diffraction peaks were also caused by the dissimilar lattice parameter for each of the crystallites present in the composite [40]. The dominant peak behavior can also be explained by the post-production having shifted the peak to a lower angle [41]. The internal strain had been unintentionally altered during the production, which may have resulted in a different degree of plastic deformation in the composite grains. High operating temperature and holding time contributed to the increase in the crystalline size. The Al_2_O_3_ also acted as a catalyst for the structural size change. Hard ceramic particles introduced into the composite caused the matrix to expand in all directions, creating better bonding in the matrix. Previous studies found that the hardness of a material increases as the crystallite size decreases [30,42,43,44]. However, their studies did not investigate the influence of heat in the process. By assisting the forming process with added heat, the crystallites increase. Kallip et al. [32] determined that the crystallite size grows when heat is supplied through the hot compaction production of the nanocomposite and that the hardness is higher when compared to cold compaction production. This is due to the pinning effect on reinforcing the constituent at the crystallite boundaries [45,46]. In short, the composite crystallite size increases not only due to the heat-assisted process, but due to Al_2_O_3_ pinned along the crystallite boundaries which yields high composite microhardness.

**Figure 5:**
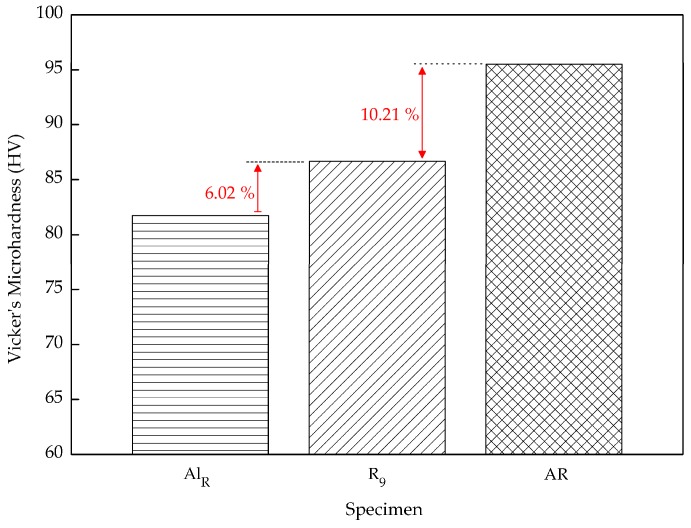
Residuals for Vickers microhardness between maximum (R_9_) parameter settings compared to the Al_R_ and AR AA6061 samples.

The residuals for Vickers microhardness, between the minimum and maximum parameter settings, in comparison with our results from unreinforced recycled AA6061 with HPF at 530 °C for 120 min, and the AR AA6061 sample are depicted in Figure 5. There is an obvious difference between the minimum and maximum parameter settings. The increase from 71.36 to 86.66 HV_0.3_ confirms that the temperature and holding time increase yielded better composite hardness. Previously, Yusuf et al. [20] recorded a hardness of 81.74 HV_0.3_ when aluminum chips were pressed at 530 °C with a 120-min holding time [47]. The increase of 6.02% is mostly due to the presence of Al_2_O_3_ particles which reinforce and strengthen the matrix and therefore increase the hardness value. As mentioned before, the hardness of AR aluminum was verified as being 95.51 HV_0.3_. When comparing the data to our present findings, the residuals were calculated at 10.21%. Initially, the atom structure in pure AL AA6061 is stable, with solid bonding, which leads to high hardness.

### 3.2. Density

**Figure 6:**
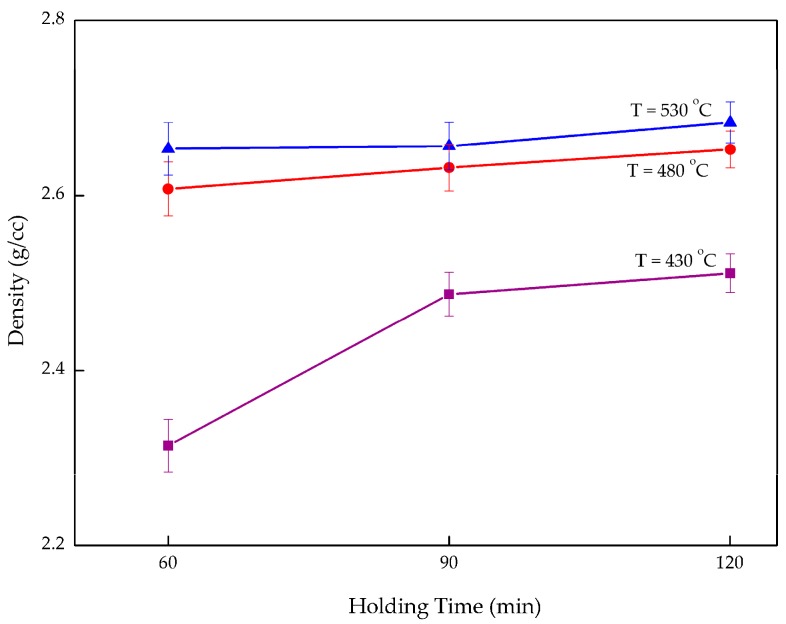
Change in density with different temperatures and holding times.

Overall, density increases with increasing holding time and the operating temperature as exhibited in Figure 6. When maintaining the temperature at 480 °C and varying holding time, the composite density increased 1.73% from 2.608 g/cc after 60 min to 2.653 g/cc after 120 min. By increasing the temperature but maintaining the holding time at 120 min, the density rose 6.85% from 2.512 g/cc at 430 °C to 2.684 g/cc at 530 °C. This again confirms that operating temperature plays a vital role in enhancing the composite performance. Insufficient temperature and holding time both affected the composite in terms of creating a large void between the chips as well as lowering the density. When exposing composites to a higher temperature, the gaps in the matrix shrink which allows Al_2_O_3_ to occupy the opening and strengthen the ductile aluminum. Increasing the holding time allowed better bonding between the chips. The right combination of high temperature and longer holding times would eventually lead to high density. In addition, the increase in the composite density may be attributed to the large difference of the initial densities [38]. The void between the chips decreases when the temperature and holding time increase. This results in it being easier for the Al_2_O_3_ particles to fill the voids and decrease the porosity, thus enhancing the density of the composite.

**Figure 7:**
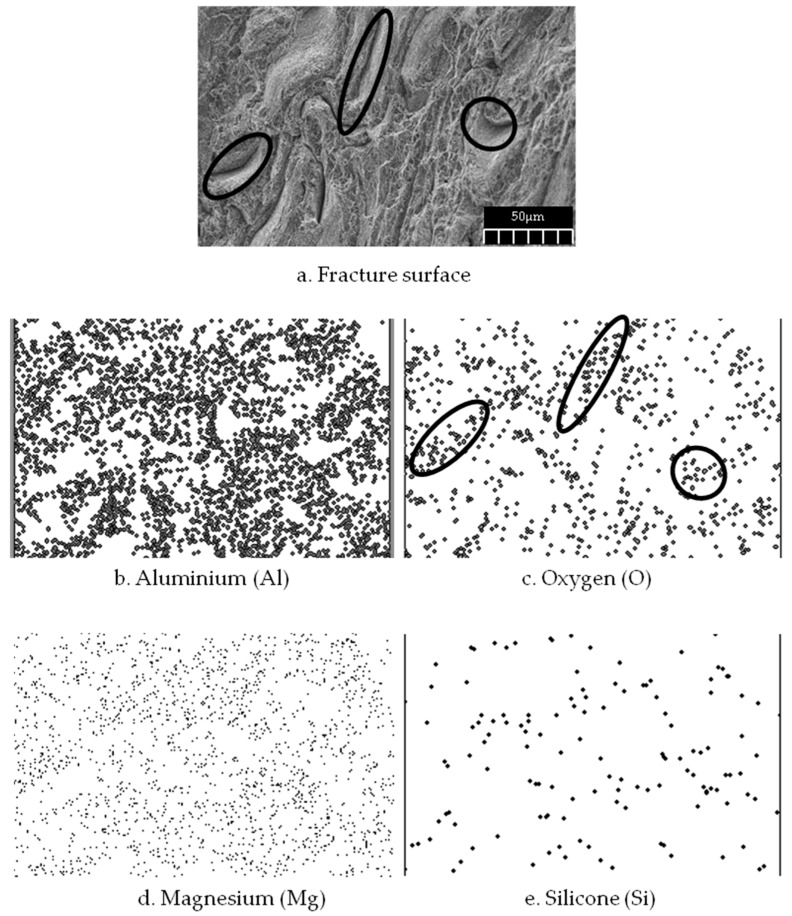
Mapping of fracture morphology of the composite.

Al_2_O_3_ contains a higher amount of oxygen compared to the initial matrix element (AA6061), which mostly contains aluminum, magnesium, and silicone. However, to confirm the composite distributions on an atomic scale, X-ray mapping was performed (Figure 7). The mapping showed that the interface is rich in Mg, Fe, and Si. The reaction between Al_2_O_3_ and the intermetallic compound leads to the formation of brittle compounds, like the MgAl_2_O_4_ spinels, which do not dissolve during solution treatment [48,49]. A fractograph of the specimen is taken at the gauge area after tensile test (Figure 7a). The mapping image shows that the mixing activity during the preparation process had uniformly distributed the Al_2_O_3_ throughout the recycled aluminum chips. In addition, the pressing action assisted by high heat was responsible for the agglomeration of Al_2_O_3_ in the gaps between the chips. The oxygen controls the distribution which means that the reinforcement particles were allocated within the gaps (Figure 7c). These void-filling activities reduced the porosity and hence caused high composite density. The Al_2_O_3_ filling of the gaps within the matrix chips is also responsible for the increase in crystallite size.

**Figure 8:**
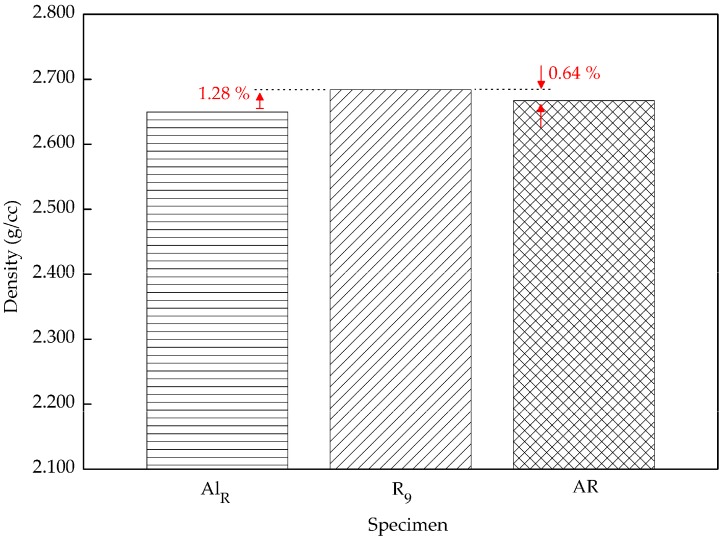
Residuals for density between the maximum (R_9_) parameter settings compared to the Al_R_ and AR AA6061 samples.

Figure 8 shows that the density has significantly increased from the lowest parameters, 60 min and 430 °C, to highest parameters, 120 min and 530 °C. Compared to the previous study by Yusuf [47], in our study the reinforcement particle increased the composite density 1.28% higher. The void-filling activities had enhanced the composite density. Furthermore, the density of the AR material is 0.64% lower than the composite. This again proved that particulate Al_2_O_3_ assists in improving the density. The density of a composite is created by the densities of the two different constituents, which are unalike. Aluminum chips have a density of 2.667 g/cc, which is ~47% lower than Al_2_O_3_, at 3.9159 g/cc. According to the mixture rule, the theoretical densities were calculated using the weight fraction of the aluminum oxide particles [22]. From the mixture rule, the calculated theoretical density is 2.692 g/cc. The value from this equation was used as a benchmark in comparing the maximum density with the theoretical density. Subsequently, the composite density was about 0.3% off of the theoretical calculation. This shows that at 530 °C and with a holding time of 120 min, paired with 2 wt % reinforcement, acceptable density can be obtained.

**Figure 9:**
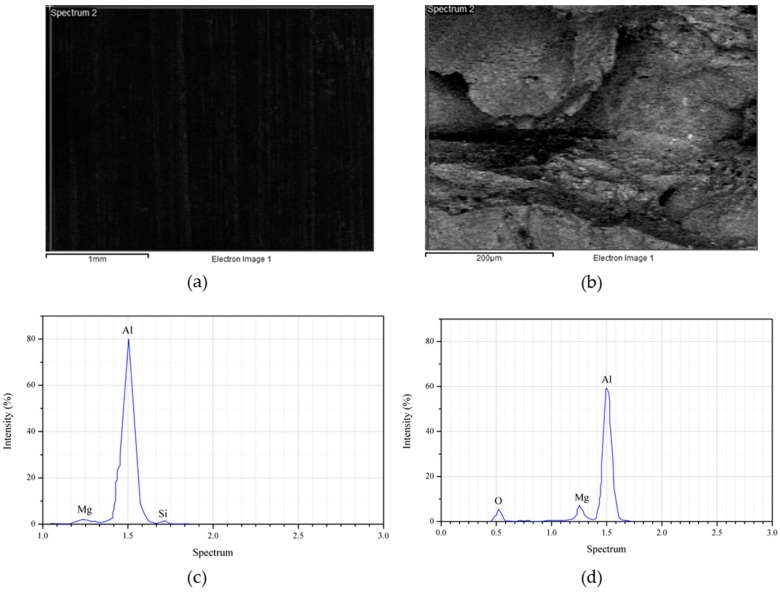
The scanning surface sample for (**a**) AR AA6061 and (**b**) MMC-Al_R_ at the maximum parameters, and the EDS spectrum for (**c**) AR AA6061 and (**d**) MMC-Al_R_ at maximum parameter.

Figure 9 depicted the EDS spectra for AR AA6061 and the reinforced aluminum waste with 2.0 wt % Al_2_O_3_ particles. The scan of the surface was taken at the cross-section of both the AR and the composite specimen, shown in Figure 9a,b respectively. The presence of Al, Mg, and Si were noticed from the peak present in the spectrum, confirming no contaminants were present (Figure 9c). When the reinforcement particle was introduced into the pure alloy, the peak changed. Additionally, the oxygen peak appeared due to high oxygen from the aluminum oxide particles (Figure 9d). On the other hand, the Si peak was hindered by the composite, which is as a result of high temperature and longer holding time. Previously, a study concluded that temperature increase leads to a refined silicon element [50]. The fine silicon spread homogeneously in the microstructure and yielded better material strength [51]. Also, the EDS and mapping analysis of the composite at maximum parameter settings indicated that the interfacial region was enriched with oxygen, especially within the composite gaps. Concisely, the reinforcement constituent helps to improve the composite performance either in terms of mechanical properties or surface integrity.

## 4. Conclusions

Formerly, recycled aluminum-based metal matrix composites were prepared using the hot press forging process with various combinations of operating temperatures and holding times. The microhardness and density of the composite were investigated and the following conclusions were drawn:

1. An increase in both microhardness and density was seen as both temperature and holding time increased. Nevertheless, temperature had a stronger effect the MMC-Al_R_ performance, compared to the holding time. The microhardness increased 4.94–10.45%, from 7.360 to 86.656 HV_0.3_, by increasing the operating temperature from 430 to 530 °C. The density increased 0.95–12.71%, from 2.314 to 2.684 g/cc, by increasing the operating temperature from 430 to 530 °C.
2. MMC-Al_R_ was much stronger than Al_R_ when compared to the AR. MMC-Al_R_ hardness is only 10.21% less, while Al_R_ had 16.85% of differentiation from AR. MMC-Al_R_ density increased 0.63%, while Al_R_ was recorded as having 0.64% of differentiation from AR.

The MMC performance was similar to the control specimen (AR). Therefore, it can be used as an alternative high-strength material and as a secondary resource to overcome the shortage of primary resources. This research can help increased environmental awareness among industrial practitioners. This approach could be an initiative to support the government in the Green Tech campaign by practicing sustainable manufacturing. This process creates new knowledge for preserving the environment from additional harm compared to the conventional method.

## Figures and Tables

**Table 1:** Initial as-received (AR) material properties.

Parameter	Nomenclature	Value
Matrix volume fraction	$f_{m}$ (%)	98.0
Reinforcement volume fraction	$f_{r}$ (%)	2.0
Matrix density	$\rho_{m}$ (g/cm^3^)	2.667
Reinforcement density	$\rho_{r}$ (g/cm^3^)	3.916
Matrix hardness	$H_{m}$ (HV)	95.512
Reinforcement hardness	$H_{r}$ (HV)	2700

**Table 2:** Selected milling parameter.

Parameter	Value	Chip Morphology
Cutting Speed, *v*	1100 m/min	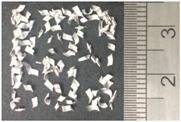
Feed, *f*	0.05 mm/tooth	
Depth of Cut, DOC	1.0 mm	

**Table 3:** Specimen designation at different operating temperatures and holding times.

Temperature (°C)	Holding Time (min)	Specimen Designation
430	60	R_1_
430	90	R_2_
430	120	R_3_
480	60	R_4_
480	90	R_5_
480	120	R_6_
530	60	R_7_
530	90	R_8_
530	120	R_9_

**Table 4:** Evolution of hardness and density with respect to different processing parameter.

Specimen	Vickers Hardness (HV_0.3_)	Density (g/cc)
R_1_	71.360	2.314
R_2_	73.367	2.487
R_3_	74.625	2.512
R_4_	76.432	2.608
R_5_	76.988	2.632
R_6_	78.454	2.653
R_7_	80.592	2.654
R_8_	83.921	2.657
R_9_	86.656	2.684
AR	95.512	2.667

**Table 5:** Grain analysis on the effect of operating temperature and holding time.

Specimen	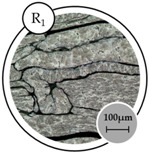	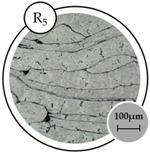	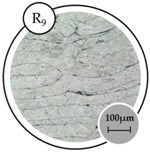
**Number of Intercepts**	95	131	202
**Mean Intercepts length (mm)**	53.28	38.64	25.05
**G number**	5.17	6.09	7.34
**Average Grain Diameter (μm)**	60.07	43.62	28.33

**Table 6:** Crystallite size of samples at peak (1,1,1).

Specimen	Peak Height (cts)	d Spacing (Å)	Crystallite Size (Å)
R_1_	14,535	2.34677	739.9
R_9_	10,452	2.34641	770.6
AR	9723	2.34527	785.5
